# Anchylosis of the Knee Joint

**Published:** 1859-03

**Authors:** 


(From the Medical and Surgical Reporter for March 5, 1859.)

*Ankylosis of the Knee-Joint — Novel Mode of Treatment by Fracture of the Thigh-Bone.*

In the case of ankylosis of the knee-joint, reported on page 390, in which the application of apparatus was temporarily suspended for fear of producing subluxation of the head of the tibia, it is proposed to-day to adopt a novel mode of treatment. Amputation is sometimes practiced in similar cases, where all other means of relief have failed, or the plan of Barton, which consists in the removal of a V-shaped section of bone, and the application of a Stromeyer's screw to straighten the limb. But the latter mode produced a sort of compound fracture of the bone, and is extremely hazardous. It is proposed to-day to bore holes in the bone, from one external orifice in the soft parts above the knee, where the bone is least covered, and after having thus weakened the part sufficiently, to break the bone either across the knee or by apparatus. This, too, is a hazardous operation, and may be followed by inflammation and its consequences, demanding amputation of the limb at a future day. But in the strong belief that the plan suggested may be of benefit, by affording hereafter an opportunity for bending the soft callus, so as to effect the straightening of the limb, the attempt will be made to fracture it. A compound fracture is not likely to result, but probably a simple form, requiring the usual simple treatment, by rest, position, splints, etc. Troublesome abscesses might follow the introduction of the ordinary gimlet, but one that will bring away the sawdust with it is preferred. The risk of the operation is subsequent necrosis, the lower end of the thigh-bone being very liable to that affection, and especially in the region which has been selected as the proper point for operating. This result may, perhaps, be avoided by a simple treatment, and by general attention to the health of the patient.

When the constitution is not good, the patient being of a scrofulous diathesis, or disease of the bone, as necrosis, is preexistent, the operation should not be thought of. If the experimental mode of treatment adopted here be successful, it will doubtless be found more efficacious than the plan of Barton, and much more humane than amputation of the limb. The gimlet should be introduced on the outside of the limb, so as to avoid the anastomotic artery. From the single external orifice, half a dozen holes were bored through the bone, at different points, and after several efforts to break the bone, it was fractured with a loud snap, distinctly audible over the whole room.

The case was exhibited at the following clinic, no unfavorable symptom having followed the operation.

### REMARKS ON THE ABOVE CASES.

These cases, treated by Drs. Eve and Pancoast, are in some respects extremely gratifying. They show that the operation proposed by Dr. Brainard, in 1854, is receiving the attention which it merits in this country, and that the award of the committee of the American Medical Association, of a prize, is fully justified by the success of the method when applied in practice. The report of the case by Prof. Eve also manifests a spirit of justice and liberality altogether in accordance with his well known character. We regret to say that the same observation is not entirely applicable to the second case. Dr. Pancoast, in the report, is made to say: "It is proposed to-day to adopt a novel mode of treatment." "It is proposed to bore holes in the bone, from one external orifice in the soft parts above the knee, where the bone is least covered, and after having thus weakened the part sufficiently, to break the bone either across the knee or by apparatus."

Now, this "novel mode" of treatment is that proposed by Dr. Brainard in his essay, published in the transactions of the American Medical Association for the year 1854, in the following terms:

"Perforation of bone may be employed to weaken it in such a manner as to cause it to fracture readily at the point desired, with but a moderate degree of force and but little pain."

"The cases in which it is proposed to apply it are,"—

1. "Perfect ankylosis by bone or fibrous adhesions too firm to be separated by moderate force, and when the member is in a position to be useless."

"This method of treatment is recommended as a substitute for the following, which are now in vogue:"

1. "The operation of Barton, which consists in making incisions, sawing out a wedge of bone," etc. "Dr. Buck has recently performed a modification of Barton's operation for bony ankylosis by resecting the articulation of the knee, an operation which I suppose may likewise be superseded by partial sub-acutaneous division of the bone."

Dr. Pancoast used a gimlet in his operation. This has been tried by Dr. Brainard, who says of it, that "it has the double inconvenience that it is liable to expose to suppuration, and that the gimlet is incapable of perforating the compact structure of bone, as any one can readily prove for himself by experience."

Dr. Pancoast, however, used a pod gimlet to bring away the sawdust. This is original. It is true, it is not difficult to make all the division requisite in this case with the instrument of Dr. Brainard, without making any dust, but if a gimlet be used, it is quite likely a pod gimlet is to be preferred.

In conclusion, we deem it but just to say that the high position of Prof. Pancoast precludes the suspicion that he should for a moment consider himself under the necessity of appropriating the ideas of others to his own use without the acknowledgment usual in such cases, but the practice, even if resorted to inadvertently, must not be allowed to grow into a habit, and thus become a precedent. We have, therefore, thought proper, at the outset, to set the subject in its true aspect, by assigning the priority there, where it is well known to belong.

